# Nurses’ preferences for interventions to improve infection prevention and control behaviors based on systems engineering initiative to patient safety model: a discrete choice experiment

**DOI:** 10.1186/s12912-024-01701-w

**Published:** 2024-01-10

**Authors:** Qian Zhou, Junjie Liu, Feiyang Zheng, Qianning Wang, Xinping Zhang, Hui Li, Li Tan, Wanjun Luo

**Affiliations:** 1 Department of Hospital Infection Management, Wuhan Children’s Hospital (Wuhan Maternal and Child Healthcare Hospital), Tongji Medical College, Huazhong University of Science and Technology , No.100 Xianggang Rd, Wuhan, Hubei Province China; 2 School of Medicine and Health Management, Tongji Medical College, Huazhong University of Science and Technology, Wuhan, Hubei China; 3 Children’s Oncology Department, Wuhan Children’s Hospital (Wuhan Maternal and Child Healthcare Hospital), Tongji Medical College, Huazhong University of Science and Technology, Wuhan, Hubei China; 4 Department of Hospital Infection Management, Tongji Hospital, Tongji Medical College, Huazhong University of Science and Technology, No.1095 Jie Fang Avenue, 430030 Hankou, Wuhan, China

**Keywords:** Behavior and behavior mechanisms, Infection control, Cross Infection, Patient safety, Latent class analysis, Ergonomics

## Abstract

**Background:**

The evidence of preferences for infection prevention and control (IPC) intervention from system perspective was lacked. This study aimed to elicit nurses’ preferences for the intervention designed to improve IPC behaviors based on the Systems Engineering Initiative to Patient Safety (SEIPS) model using Discrete Choice Experiment (DCE).

**Methods:**

A DCE was conducted among nurses who were on active duty and willing to participate from July 5th to 10th, 2021 in a tertiary hospital in Ganzhou City, Jiangxi Province, using convenience sampling. A self-administered questionnaire included scenarios formed by six attributes with varying levels based on SEIPS model: person, organization, tools and technology, tasks, internal environment and external environment. A conditional logit and latent class logit model were performed to analyze the data.

**Results:**

A total of 257 valid questionnaires were analyzed among nurses. The results from the latent class logit model show that nurses’ preferences can be divided into three classes. For nurses in multifaceted-aspect-preferred class (41.9%), positive coefficients were obtained in those six attributes. For person-preferred class (19.7%), only person was positively significant. For environment-preferred class (36.4%), the most important attribute were tasks, tools and technology, internal environment and external environment.

**Conclusions:**

This finding suggest that nurses have three latent-class preferences for interventions. Multifaceted interventions to improve IPC behaviors based on the SEIPS model are preferred by most nurses. Moreover, relevant measured should be performed targeted the latent class of person-preferred and external-environment-preferred nurses.

**Supplementary Information:**

The online version contains supplementary material available at 10.1186/s12912-024-01701-w.

## Background

Healthcare-associated infections (HCAI) have become a major threat to global health, leading to the spread of drug-resistant organisms, prolonged hospital stays, long-term disability, increased mortality rates, and additional costs [1]. It is estimated that HCAI resulted in an increase of 13.89 days of hospitalization, an increase of 24881.37 average medical cost and an increase of 9438.46 average drug cost [2]. By implementing infection prevention and control (IPC) behaviors such as physical distancing, mask-wearing, and eye protection, the risk of HCAI can be greatly reduced, potentially leading to 55–70% fewer cases of HCAI [3, 4].

Previous studies have investigated the factors that influence IPC behavior in healthcare workers, primarily from a person-centered perspective [5, 6]. For example, subjective norm, subjective norm and perceived behavioral control are associated with hand hygiene behaviors according to theory of planned behavior [7]. Nevertheless, IPC is a complex system that entails numerous system-related components, including the individuals, workload, and the tools used and environment, etc. [5, 8–10]. Each of these system-related elements may pose risks for HCAI acquisition, making it essential to take into account all the system-related factors with respect to improve IPC behaviors. Unfortunately, most studies fail to incorporate critical system-related factors in the analysis of IPC process simultaneously, leaving out the relevant analysis and resulting in incomplete findings [11]. Some studies have attempted theoretical explorations of IPC behaviors in healthcare workers from a systems-oriented perspective, such as normalization process theory and non-representational theory [12, 13]. However, there is a lack of an explicit definition of key components or pathway regarding IPC mechanism [14]. Consequently, a system-related pathway exploration to fully understand the mechanisms of IPC.

Systems Engineering Initiative to Patient Safety (SEIPS) model, developed specifically for healthcare to improve patient safety and medical quality through examining the mechanism in healthcare systems comprehensively [15]. An updated version of the SEIPS model, SEIPS 2.0, identifies specific structural components including person, organization, tools and technology, tasks, internal environment, and external environment and their contribution to the healthcare process and outcomes [16]. Our study chose the SEIPS 2.0 version as it is more appropriate for analyzing the impact of system-related factors on IPC behaviors among healthcare workers, while SEIPS 3.0 is more suitable for the exploration of healthcare transition [17]. The SEIPS model offers numerous benefits when applied to IPC. First, it has the potential to identify the principal contributors and barriers to IPC issues. Second, it provides a theoretical foundation for promoting IPC behavior from a system perspective, with its distinct elements and interrelatedness. Finally, it considers numerous broad outcomes and attaches equal importance to non-human factors, which constitutes a considerable advantage to solve system problem [18, 19]. However, there remains a significant dearth of quantitative evidence in the SEIPS model with regard to IPC-related problems.

Previous studies have not adequately addressed the evidence of nurses’ preference for IPC interventions. Nurses play a pivotal role in implementing IPC as the largest group of practitioners, and their high-level compliance with IPC behaviors is crucial in preventing the spread of HCAI, with the significant differences in the nature and social structure of nursing work compared to medicine and allied health professions [20]. Meanwhile, researchers can analyze how individuals’ preferences differ for each attribute and how the interaction of various elements affects their decision-making using discrete choice experiment (DCE). Within the context of enhancing IPC, this study aimed to utilize DCE to capture nurses’ preferences for interventions targeted at improving their IPC behaviors based on the SEIPS model. Obtaining such insight can provide effective support for interventions in the perspective of IPC system, while the further application of latent class analysis may facilitate the understanding of any variations that exist across different groups in their preferences to promote IPC behaviors among nurses.

## Methods

### Participants

The participants in this study were front-line nurses who were recruited from a tertiary hospital located in Ganzhou City, Jiangxi Province. The hospital is equipped with approximately 3,000 beds and ranks in the top three in performance assessments among similar hospitals in Jiangxi Province. We conducted a cross-sectional face-to-face questionnaire survey by convenience sampling from July 5th to 10th, 2021, among nurses who were on active duty and willing to participate.

The inclusion criteria included ① front-line nurses working in the clinic, ②having a minimum of one year of clinical work experience. The exclusion criteria encompassed ①interns, ②training staff. The sample size required was determined based on the number of choice tasks (t), the number of alternatives (a), and the largest number of levels for the attributes (c) using the equation N > 500c / (t × a) [21].According to the equation, the sample size of this study should be greater than 31.25.

### Attributes and levels

DCE is a method that involves hypothetical scenarios to systematically investigate nurses’ preferences for interventions. To identify factors that influence IPC behaviors based on the SEIPS model, we conducted a literature review. Subsequently, we conducted a qualitative interview involving 18 expert healthcare workers (11 nurses, 5 doctors, and 2 infection preventionists) to confirm the attributes and levels based on the perspectives of stakeholders. The literature review and qualitative interviews informed the determination of the attributes and levels of the intervention based on the SEIPS model [22]. Six attributes were determined: person, organization, tools and technology, tasks, internal environment, and external environment. Overall. all attributes had two levels, except for IPC tasks, which had three levels (Table 1).

**Table 1:** Intervention attributes and levels

Attribute	Definition	Level
Person	Knowledge and attitude of nurses	Better IPC knowledge and awareness training
		No measure
Organization	Activities of IPC organization and management	IPC organization improvement (strengthen leaders’ attention and cultivate a safe atmosphere about IPC)
		No measure
Tools and technology	Quality and comfort of protective equipment and the impact of protective equipment use on nurses	Improvement of the availability and comfort of protective equipment
		No measure
Tasks	The workload of IPC tasks and conflicts with other tasks	IPC workflow improvement
		Reduced workload
		No measure
Internal environment	The physical environment of the department where nurses work and perform IPC behaviors	Physical environment improvement (department layout, number and location of hand hygiene facilities, etc.)
		No measure
External environment	Influence factors outside the organization and IPC-related policies	External environment improvement (medical policies support and social positive media)
		No measure

### Questionnaire design and quality control

The D-efficiency design is the most efficient and widely used partial factorial design nowadays, which can ensure that each attribute is independent, and the different levels of each attribute appear at the same frequency. In this study, D-efficiency was designed by Ngene software generating 24 combinations in 2 blocks, with 12 combinations for each version. The survey was structured as follows (supplementary file): the aim and description of the research, a practice question with explanation, discrete choice questions (Table 2) and socio-demographic character: gender, age, work year, degree, title and department.

**Table 2:** Example choice scenario

	Scenario A	Scenario B
Person (For example, IPC knowledge and awareness training)	Better IPC knowledge and awareness training	No measure
Organization (For example, attention to IPC of leaders and cultivating a safe atmosphere)	No measure	IPC organization improvement
Tools and technology (For example, quality and comfort of protective equipment)	No measure	Improvement of the availability and comfort of protective equipment
Tasks (For example, workload and conflicts with other tasks)	IPC workflow improvement	Reduced workload
Internal environment (For example, department layout, number and location of hand hygiene facilities, etc.)	Physical environment improvement	No measure
External environment (For example, social respect and understanding, policy support)	No measure	External environment improvement
Please choose your most preferred scenario:	I prefer Scenario A ☐	I prefer Scenario B ☐

In terms of quality control in the data collection, this study implemented several measures. Firstly, a repeated design of a selection set was adopted to test the consistency of respondents’ answers. The questionnaire included 13 combination sets, with the 5th combination set repeated as the 13th combination set for validation purposes. Additionally, to ensure the survey’s clarity and ease of understanding for nurses, it underwent a pilot test with 20 clinical nurses. Based on their feedback, any inappropriate or confusing information was revised to enhance comprehension and interpretation. Furthermore, to ensure that respondents carefully read the scenarios, the questionnaire was administered in the presence of trained investigators.

### Data analysis

Descriptive statistics were used to describe the characteristics of participants. We used a conditional logit model, which is a commonly-used DCE model derived by McFadden based on economic theory, to determine the average utility assigned to the attribute level compared to the reference level in the preference among nurses. Since the conditional logit model ignores preference heterogeneity, we employed a latent class logit model to further explore the potential classes in preference, as the latent class of preference is commonly seen. The latent analysis can examine both the homogeneous preferences within classes and heterogeneous preferences across classes [23]. The number of the latent classes was determined by comparing the model fitness in the model with 2, 3, 4, 5 and 6 classes. All levels were treated as categorical variables with the level of no measure taken as the reference, while dummy coding was applied for three attribute levels in the model. We considered p < 0.05 as the threshold for statistical significance, indicating that an attribute level is significantly different from that of the reference level. A positive coefficient indicates that an attribute level is preferred over the reference level in the intervention scenario. All the analyses were performed using Stata 15.0 (StataCorp LP, College Station, TX, USA).

## Results

### Participants’ characteristics

The response rate was 93.36%, with 301 questionnaires distributed and 281 valid questionnaires obtained. Finally, 259 questionnaires were included in the analysis, and 22 were excluded because of failing to pass logical consistency. Among the 259 survey respondents, most were women (256, 98.94%). The vast majority of nurses were younger than 45 years old (245, 94.59%), worked for less than 20 years (229, 88.42%), held intermediate or lower professional titles (232, 89.58%), and most had bachelor’s degrees (252, 97.30%) (Table 3).

**Table 3:** Participants’ characteristics

	Category	N	Percentage (%)
gender			
	male	3	1.16
	female	256	98.84
Age ^a^			
	≤ 29	95	36.68
	30–44	150	57.92
	≥ 45	13	5.02
work year ^a^			
	≤ 9	128	49.42
	10–19	101	39.00
	≥ 20	27	10.42
Degree ^a^			
	Master and above	1	0.39
	Undergraduate	208	80.31
	junior college and below	44	16.99
Title ^a^			
	No title	14	5.41
	Junior	112	43.24
	Intermediate	106	40.93
	Deputy senior and above	22	8.49
Department ^a^			
	Internal medicine	90	34.75%
	Surgery medicine	85	32.82%
	Others	80	30.89%

^a^: One questionnaire missed age, three missed work year, six missed degree, five missed title, and four missed department

### Discrete choice experiment results: conditional logit model

From the results of the conditional logit model, person factor was the most valued in the attribute of IPC intervention. Nurses preferred interventions featured better IPC knowledge and awareness training (*β* = 0.797, *P* < 0.001) compared to interventions with no measures adopted. In addition, interventions were preferred when improvement of IPC organization and management (*β* = 0.318, *P* < 0.001), improvement of the availability and comfort of protective equipment (*β* = 0.475, *P* < 0.001), improvement of IPC workflow (*β* = 0.382, *P* < 0.001), reduced workload (*β* = 0.337, *P* < 0.001) and improvement of the physical (*β* = 0.450, *P* < 0.001) and external environment (*β* = 0.614, *P* < 0.001) were adopted (Table 4).

**Table 4:** Conditional logit model result of preference

	β	Standard error	Z	*P*-value	95% Confidence interval
Person (Better IPC knowledge and awareness training)	0.797	0.073	10.88	< 0.001	(0.653, 0.940)
Organization (IPC organization improvement)	0.318	0.045	7.11	< 0.001	(0.230, 0.405)
Tools and technology (Improvement of The availability and comfort of protective equipment)	0.475	0.044	10.76	< 0.001	(0.388, 0.561)
Tasks (IPC workflow improvement)	0.382	0.058	6.55	< 0.001	(0.268, 0.497)
Tasks (Reduced workload)	0.337	0.063	5.31	< 0.001	(0.212, 0.461)
Internal environment (Physical environment improvement)	0.450	0.055	8.20	< 0.001	(0.343, 0.558)
External environment (External environment improvement)	0.614	0.053	11.67	< 0.001	(0.511, 0.717)

### Discrete choice experiment results: latent class logit model

The results of the latent class logit model suggest that three classes are best suited to capture the preferences of nurses regarding IPC interventions based on the model parsimony and model fit. The largest proportion, comprising 41.9% of nurses, belonged to class one, which is referred to as the multifaceted-aspect-preferred class. Nurses in class one preferred the intervention that focused on better IPC knowledge and awareness training (*β* = 1.071, *P* < 0.001), IPC organization improvement (*β* = 0.799, *P* < 0.001), improvement of the availability and comfort of protective equipment (β = 0.955, P < 0.001), IPC workflow improvement (*β* = 0.804, *P* < 0.001), reduced workload (*β* = 0.326, *P* = 0.015), IPC physical environment improvement (*β* = 0.735, *P* < 0.001) and IPC external environment improvement (*β* = 0.603, *P* < 0.001). In general, nurses in class one preferred to address multiple aspects of IPC practice to create a comprehensive and effective intervention (Table 5).

**Table 5:** Latent class logit model result of preference for each latent class

	*β*	Standard error	Z	*P*-value	95% Confidence interval
Class one					
Person (Better IPC knowledge and awareness training)	1.071	0.131	8.200	< 0.001	(0.815, 1.327)
Organization (IPC organization improvement)	0.799	0.141	5.660	< 0.001	(0.522, 1.076)
Tools and technology (Improvement of The availability and comfort of protective equipment)	0.955	0.120	7.980	< 0.001	(0.720, 1.189)
Tasks (IPC workflow improvement)	0.804	0.155	5.190	< 0.001	(0.500, 1.108)
Tasks (Reduced workload)	0.326	0.135	2.420	0.015	(0.062, 0.591)
Internal environment (Physical environment improvement)	0.735	0.103	7.150	< 0.001	(0.553, 0.936)
External environment (External environment improvement)	0.603	0.113	5.320	< 0.001	(0.381, 0.825)
Class two					
Person (Better IPC knowledge and awareness training)	3.770	0.431	8.740	< 0.001	(2.925, 4.616)
Organization (IPC organization improvement)	-0.075	0.293	-0.250	0.723	(-0.648,0.499)
Tools and technology (Improvement of The availability and comfort of protective equipment)	0.281	0.341	0.820	0.529	(-0.388,0.951)
Tasks (IPC workflow improvement)	0.220	0.415	0.530	0.213	(-0.594,1.033)
Tasks (Reduced workload)	0.018	0.460	0.040	0.889	(-0.883, 0.919)
Internal environment (Physical environment improvement)	-0.535	0.363	-1.470	0.160	(-1.247,0.178)
External environment (External environment improvement)	0.167	0.340	0.490	0.863	(-0.500,0.834)
Class three					
Person (Better IPC knowledge and awareness training)	-0.056	0.118	-0.480	0.509	(-0.287,0.175)
Organization (IPC organization improvement)	0.044	0.078	0.570	0.853	(-0.108,0.197)
Tools and technology (Improvement of the availability and comfort of protective equipment)	0.269	0.081	3.310	0.001	(0.110,0.429)
Tasks (IPC workflow improvement)	0.252	0.110	2.280	0.030	(0.035,0.468)
Tasks (Reduced workload)	0.600	0.121	4.960	< 0.001	(0.363,0.837)
Internal environment (Physical environment improvement)	0.415	0.079	5.240	< 0.001	(0.260,0.571)
External environment (External environment improvement)	0.962	0.101	9.550	< 0.001	(0.765,1.160)

The results of the latent class logit model indicate that 19.7% of nurses were in class two, which is referred to as the person-preferred class. This class had a strong preference for IPC knowledge and awareness training, with the coefficient of the person factor (better IPC knowledge and awareness training) being highly significant (*β* = 3.767, *P* < 0.001) while the coefficients for other factors were not significant. This suggests that nurses in this class place a great emphasis on the importance of training as a means to improve IPC behaviors.

**Fig. 1:**
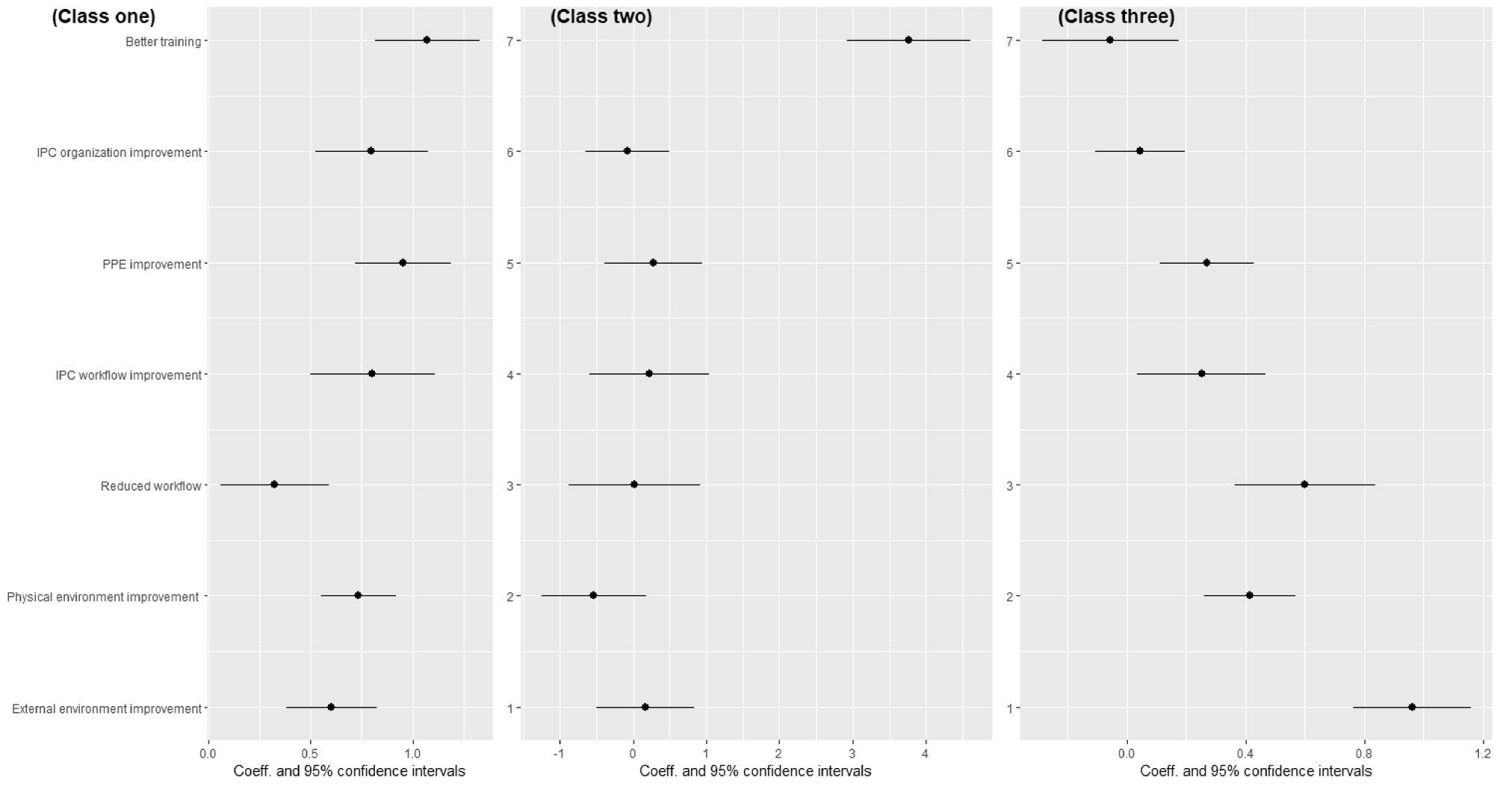
Latent class logit model result of preference for each latent class

According to the results of the latent class logit model, 36.4% of nurses fell into class three, which is referred to as the environment-preferred class. Nurses in this class placed high value on interventions that characterized as improvement of the availability and comfort of protective equipment (*β* = 0.268, *P* = 0.001), IPC workflow improvement (*β* = 0.252, *P* = 0.030), reduced workload (*β* = 0.600, *P* < 0.001), IPC physical environment improvement (*β* = 0.415, *P* < 0.001) and IPC external environment improvement (*β* = 0.962, *P* < 0.001). However, unlike the characteristics above, person (better IPC knowledge and awareness training) (*β*=-0.056, *P* = 0.509) and organization (IPC organization improvement) (*β* = 0.044, *P* = 0.853) had little influence on nurses’ willingness to choose interventions. Nurses in this class tended to adopt the intervention with external environment improvement mostly (Fig. 1).

## Discussion

This study explored nurses’ preferences for the intervention designed to improve IPC behaviors based on the SEIPS model. Our results suggested the heterogeneity among nurses in the preferences of interventions for IPC behaviors. In addition to the multifaceted-aspect-preferred class, nurses can also be categorized into person-preferred class and environment-preferred class.

The latent class logit model used in this study demonstrated superior fitness to the data, handling preference heterogeneity and information richness more effectively than the conditional logit models. The analysis revealed three distinct classes of nurses with different preferences for IPC behavior interventions.

The majority of nurses, belonging to multifaceted-aspect-preferred class, preferred interventions that comprehensively improve person, organization, technology and tools, task, internal environment, and external environment factors in the SEIPS model, which are consistent with previous studies [24–26]. The key attributes of the intervention strategy involving several aspects of the IPC work system in the SEIPS model, such as person, organization, tool and technology, task, internal environment and external environment factors, have been highlighted in previous studies as essential components in successful interventions. For example, Gould et al. identified the importance of interventions that improve the internal environment, such as increasing the availability of hand hygiene consumption, training through different types of education, and organizational support [9], while McAteer et al. emphasize the significance of the improvement of IPC tasks by providing designated time for IPC tasks and appropriate assignment of IPC tasks based on qualitative research [27]. Personal factors such as capacity, knowledge, and attitude play a crucial role in successful IPC practices, while organizational factors such as safety atmosphere, organizational commitment, and leadership are essential in promoting a culture of infection prevention [28]. Improving technology and tools, such as hand hygiene equipment, and the comfort of personal protective equipment also contribute to better adherence to IPC practices [26]. Finally, the design of tasks [5], and the internal and external environment of clinical settings also impact the effectiveness of IPC practices [25, 29]. The results of this study add to the existing literature by providing further evidence that the intervention approach in line with the SEIPS model can be a successful strategy for improving IPC behaviors among nurses. By addressing multiple aspects of the IPC work system, the intervention that addresses the needs and preferences of different nurses can effectively encourage the compliance to IPC practices, and ultimately improve patient outcomes.

Class two (person-preferred class), comprising the smallest number of nurses, demonstrates a strong inclination towards IPC behavior training as a means to enhance their infection prevention and control practices. The fact that this class had no preference in other attribute indicates that they may not prioritize other attributes of intervention as much as training. This finding corresponds to previous studies where some nurses believed that their poor IPC behaviors attribute to lack of knowledge about their significance and the consequences of not following them [4]. Furthermore, due to limited knowledge about IPC among many nurses, training may be the primary approach to enhance their knowledge and attitudes towards IPC that they can readily acknowledge. Consequently, there is a tendency for nurses to prioritize this attribute when making choices.

The findings of this study suggest that approximately one-third of nurses (class three), belonging to environment-preferred class, have no differences in the preference of interventions that focus on IPC knowledge and awareness training or IPC organization improvement. These nurses instead prioritize external factors, such as tools and technology, task, internal environment and external environment. This preference is in accordance with the principles of the human factors approach, which emphasizes the importance of considering the system as a whole rather than attributing errors solely to individuals [30]. In addition, the non-selection of training and management in nurses may be explained by the excessive workload and demands on their time due to the training [31]. Frequent or complex training and examinations can often lead to fatigue among nurses, posing as an obstacle to improving IPC behaviors. Healthcare organizations should strive to provide appropriate training and management to support nurses in their IPC practices. Meanwhile, by addressing the external factors, healthcare organizations can ensure that nurses have the resources and tools needed to carry out effective IPC practices.

### Implication

This study used DCE to quantify the preferences of nurses for IPC behavior improvement strategies and provide a useful reference for hospital managers and policymakers in designing and implementing effective IPC behavior improvement strategies. The classification of nurses into different classes suggests that adjustments need to be made based on the differing preferences when devising intervention strategies. To better address the needs of nurses, intervention strategies should comprehensively consider factors related to the SEIPS model. These factors include enhancing IPC knowledge and awareness through training, improving IPC organization, ensuring the availability and comfort of protective equipment, optimizing IPC workflow, reducing workload, enhancing the physical environment for IPC, and addressing external environmental factors. Furthermore, in cases where interventions may not yield optimal results, it is important to incorporate preference-based measures tailored to this population of nurses. Measures such as targeted IPC training, reducing training and examination pressures, or enhancing the training or work environment can be explored to ensure the effectiveness of interventions. In the future, it would be valuable to further explore the characteristics and influencing factors of latent classes among nurses.

### Limitations

Although we tried our best to investigate nurses who were on duty to ensure the data are representative, participants in this study were recruited from one tertiary hospital, which potentially could limit the generalizability and application of the study’s findings to other healthcare settings. In the future, this model can be further evaluated among primary medical staff to ensure that it is generalizable across different healthcare contexts.

## Conclusions

The study’s findings identified three classes of nurses with different preferences for IPC behavior interventions. Most nurses exhibited a preference for comprehensive interventions that considered various factors related to the SEIPS model. Apart from multifaceted-aspect-preferred class, nurses can also be divided into person-preferred class and environment-preferred class. It is, therefore, essential to perform preference-based measures in different classes to ensure the acceptability and effectiveness of the intervention. This approach can help tailor the IPC intervention strategies to the specific needs of each class of nurses, thereby ensuring its effectiveness and sustainability.

### Electronic supplementary material

Below is the link to the electronic supplementary material.

Supplementary Material 1

## Data Availability

The data that support the findings of this study are available from surveyed local institutions but restrictions apply to the availability of these data, which were used under license for the current study, and so are not publicly available. Data are however available from the authors upon reasonable request and with permission of surveyed local institutions.

## Abbreviations

SEIPS: Systems Engineering Initiative to Patient Safety
IPC: infection prevention and control
HCAI: healthcare-associated infections
DCE: Discrete Choice Experiment
