# Supplementary material for: Nurses’ preferences for interventions to improve infection prevention and control behaviors based on systems engineering initiative to patient safety model: a discrete choice experiment
Source: BMC Nurs. 2024 Jan 10;23:29. doi: 10.1186/s12912-024-01701-w (PMC10777601; doi:10.1186/s12912-024-01701-w)
Supplement: Supplementary file 1 — Supplementary Material 1 [file 12912_2024_1701_MOESM1_ESM.docx]

**Supplementary material**

**Block one**

**Part one: Preference of intervention**

You can choose the preferred intervention scenario from two alternatives in one set. Each scenario is formulated by the combination of the six attributes in different level. You should compare the six attributes comprehensively and then make your choice. Thirteen sets were formed in this questionnaire.

- **Sample：**

If you choose scenario A, it means that you are more willing to choose scenario A as an intervention to improve your infection prevention and control (IPC) behavior than scenario B.

|  | Scenario A | Scenario B |
| --- | --- | --- |
| Person (For example, IPC knowledge and awareness training) | No measure | Better IPC knowledge and awareness training |
| Organization (For example, attention to IPC of leaders and cultivating a safe atmosphere) | IPC organization improvement | No measure |
| Tools and technology (For example, quality and comfort of protective equipment) | Improvement of the availability and comfort of protective equipment | No measure |
| Tasks (For example, workload and conflicts with other tasks) | No measure | Reduced workload |
| Internal environment (For example, department layout, number and location of hand hygiene facilities, etc.) | Physical environment improvement | No measure |
| External environment (For example, social respect and understanding, policy support) | No measure | External environment improvement |
| Please choose your most preferred scenario: | I prefer Scenario A  ☑ | I prefer Scenario B  □ |

- **Formal Set**
- Set 1

|  | Scenario A | Scenario B |
| --- | --- | --- |
| Person (For example, IPC knowledge and awareness training) | No measure | Better IPC knowledge and awareness training |
| Organization (For example, attention to IPC of leaders and cultivating a safe atmosphere) | No measure | IPC organization improvement |
| Tools and technology (For example, quality and comfort of protective equipment) | No measure | Improvement of the availability and comfort of protective equipment |
| Tasks (For example, workload and conflicts with other tasks) | No measure | IPC workflow improvement |
| Internal environment (For example, department layout, number and location of hand hygiene facilities, etc.) | No measure | Physical environment improvement |
| External environment (For example, social respect and understanding, policy support) | No measure | External environment improvement |
| Please choose your most preferred scenario: | I prefer Scenario A | I prefer Scenario B  □ |

- Set 2

|  | Scenario A | Scenario B |
| --- | --- | --- |
| Person (For example, IPC knowledge and awareness training) | Better IPC knowledge and awareness training | No measure |
| Organization (For example, attention to IPC of leaders and cultivating a safe atmosphere) | IPC organization improvement | No measure |
| Tools and technology (For example, quality and comfort of protective equipment) | No measure | Improvement of the availability and comfort of protective equipment |
| Tasks (For example, workload and conflicts with other tasks) | Reduced workload | No measure |
| Internal environment (For example, department layout, number and location of hand hygiene facilities, etc.) | No measure | Physical environment improvement |
| External environment (For example, social respect and understanding, policy support) | No measure | External environment improvement |
| Please choose your most preferred scenario: | I prefer Scenario A | I prefer Scenario B  □ |

- Set 3

|  | Scenario A | Scenario B |
| --- | --- | --- |
| Person (For example, IPC knowledge and awareness training) | Better IPC knowledge and awareness training | No measure |
| Organization (For example, attention to IPC of leaders and cultivating a safe atmosphere) | No measure | IPC organization improvement |
| Tools and technology (For example, quality and comfort of protective equipment) | Improvement of the availability and comfort of protective equipment | No measure |
| Tasks (For example, workload and conflicts with other tasks) | IPC workflow improvement | Reduced workload |
| Internal environment (For example, department layout, number and location of hand hygiene facilities, etc.) | No measure | Physical environment improvement |
| External environment (For example, social respect and understanding, policy support) | No measure | External environment improvement |
| Please choose your most preferred scenario: | I prefer Scenario A | I prefer Scenario B  □ |

- Set 4

|  | Scenario A | Scenario B |
| --- | --- | --- |
| Person (For example, IPC knowledge and awareness training) | Better IPC knowledge and awareness training | No measure |
| Organization (For example, attention to IPC of leaders and cultivating a safe atmosphere) | IPC organization improvement | No measure |
| Tools and technology (For example, quality and comfort of protective equipment) | Improvement of the availability and comfort of protective equipment | No measure |
| Tasks (For example, workload and conflicts with other tasks) | IPC workflow improvement | Reduced workload |
| Internal environment (For example, department layout, number and location of hand hygiene facilities, etc.) | No measure | Physical environment improvement |
| External environment (For example, social respect and understanding, policy support) | No measure | External environment improvement |
| Please choose your most preferred scenario: | I prefer Scenario A | I prefer Scenario B  □ |

- Set 5

|  | Scenario A | Scenario B |
| --- | --- | --- |
| Person (For example, IPC knowledge and awareness training) | No measure | Better IPC knowledge and awareness training |
| Organization (For example, attention to IPC of leaders and cultivating a safe atmosphere) | No measure | IPC organization improvement |
| Tools and technology (For example, quality and comfort of protective equipment) | No measure | Improvement of the availability and comfort of protective equipment |
| Tasks (For example, workload and conflicts with other tasks) | No measure | IPC workflow improvement |
| Internal environment (For example, department layout, number and location of hand hygiene facilities, etc.) | Physical environment improvement | No measure |
| External environment (For example, social respect and understanding, policy support) | No measure | External environment improvement |
| Please choose your most preferred scenario: | I prefer Scenario A | I prefer Scenario B  □ |

- Set 6

|  | Scenario A | Scenario B |
| --- | --- | --- |
| Person (For example, IPC knowledge and awareness training) | No measure | Better IPC knowledge and awareness training |
| Organization (For example, attention to IPC of leaders and cultivating a safe atmosphere) | IPC organization improvement | No measure |
| Tools and technology (For example, quality and comfort of protective equipment) | Improvement of the availability and comfort of protective equipment | No measure |
| Tasks (For example, workload and conflicts with other tasks) | Reduced workload | No measure |
| Internal environment (For example, department layout, number and location of hand hygiene facilities, etc.) | Physical environment improvement | No measure |
| External environment (For example, social respect and understanding, policy support) | No measure | External environment improvement |
| Please choose your most preferred scenario: | I prefer Scenario A | I prefer Scenario B  □ |

- Set 7

|  | Scenario A | Scenario B |
| --- | --- | --- |
| Person (For example, IPC knowledge and awareness training) | Better IPC knowledge and awareness training | No measure |
| Organization (For example, attention to IPC of leaders and cultivating a safe atmosphere) | No measure | IPC organization improvement |
| Tools and technology (For example, quality and comfort of protective equipment) | No measure | Improvement of the availability and comfort of protective equipment |
| Tasks (For example, workload and conflicts with other tasks) | No measure | IPC workflow improvement |
| Internal environment (For example, department layout, number and location of hand hygiene facilities, etc.) | No measure | Physical environment improvement |
| External environment (For example, social respect and understanding, policy support) | External environment improvement | No measure |
| Please choose your most preferred scenario: | I prefer Scenario A | I prefer Scenario B  □ |

- Set 8

|  | Scenario A | Scenario B |
| --- | --- | --- |
| Person (For example, IPC knowledge and awareness training) | No measure | Better IPC knowledge and awareness training |
| Organization (For example, attention to IPC of leaders and cultivating a safe atmosphere) | IPC organization improvement | No measure |
| Tools and technology (For example, quality and comfort of protective equipment) | Improvement of the availability and comfort of protective equipment | No measure |
| Tasks (For example, workload and conflicts with other tasks) | IPC workflow improvement | Reduced workload |
| Internal environment (For example, department layout, number and location of hand hygiene facilities, etc.) | No measure | Physical environment improvement |
| External environment (For example, social respect and understanding, policy support) | External environment improvement | No measure |
| Please choose your most preferred scenario: | I prefer Scenario A | I prefer Scenario B  □ |

- Set 9

|  | Scenario A | Scenario B |
| --- | --- | --- |
| Person (For example, IPC knowledge and awareness training) | No measure | Better IPC knowledge and awareness training |
| Organization (For example, attention to IPC of leaders and cultivating a safe atmosphere) | No measure | IPC organization improvement |
| Tools and technology (For example, quality and comfort of protective equipment) | No measure | Improvement of the availability and comfort of protective equipment |
| Tasks (For example, workload and conflicts with other tasks) | IPC workflow improvement | Reduced workload |
| Internal environment (For example, department layout, number and location of hand hygiene facilities, etc.) | Physical environment improvement | No measure |
| External environment (For example, social respect and understanding, policy support) | External environment improvement | No measure |
| Please choose your most preferred scenario: | I prefer Scenario A | I prefer Scenario B  □ |

- Set 10

|  | Scenario A | Scenario B |
| --- | --- | --- |
| Person (For example, IPC knowledge and awareness training) | Better IPC knowledge and awareness training | No measure |
| Organization (For example, attention to IPC of leaders and cultivating a safe atmosphere) | IPC organization improvement | No measure |
| Tools and technology (For example, quality and comfort of protective equipment) | No measure | Improvement of the availability and comfort of protective equipment |
| Tasks (For example, workload and conflicts with other tasks) | Reduced workload | No measure |
| Internal environment (For example, department layout, number and location of hand hygiene facilities, etc.) | Physical environment improvement | No measure |
| External environment (For example, social respect and understanding, policy support) | External environment improvement | No measure |
| Please choose your most preferred scenario: | I prefer Scenario A | I prefer Scenario B  □ |

- Set 11

|  | Scenario A | Scenario B |
| --- | --- | --- |
| Person (For example, IPC knowledge and awareness training) | No measure | Better IPC knowledge and awareness training |
| Organization (For example, attention to IPC of leaders and cultivating a safe atmosphere) | No measure | IPC organization improvement |
| Tools and technology (For example, quality and comfort of protective equipment) | Improvement of the availability and comfort of protective equipment | No measure |
| Tasks (For example, workload and conflicts with other tasks) | Reduced workload | No measure |
| Internal environment (For example, department layout, number and location of hand hygiene facilities, etc.) | Physical environment improvement | No measure |
| External environment (For example, social respect and understanding, policy support) | External environment improvement | No measure |
| Please choose your most preferred scenario: | I prefer Scenario A | I prefer Scenario B  □ |

- Set 12

|  | Scenario A | Scenario B |
| --- | --- | --- |
| Person (For example, IPC knowledge and awareness training) | Better IPC knowledge and awareness training | No measure |
| Organization (For example, attention to IPC of leaders and cultivating a safe atmosphere) | IPC organization improvement | No measure |
| Tools and technology (For example, quality and comfort of protective equipment) | Improvement of the availability and comfort of protective equipment | No measure |
| Tasks (For example, workload and conflicts with other tasks) | No measure | IPC workflow improvement |
| Internal environment (For example, department layout, number and location of hand hygiene facilities, etc.) | Physical environment improvement | No measure |
| External environment (For example, social respect and understanding, policy support) | External environment improvement | No measure |
| Please choose your most preferred scenario: | I prefer Scenario A | I prefer Scenario B  □ |

- Set 13

|  | Scenario A | Scenario B |
| --- | --- | --- |
| Person (For example, IPC knowledge and awareness training) | No measure | Better IPC knowledge and awareness training |
| Organization (For example, attention to IPC of leaders and cultivating a safe atmosphere) | No measure | IPC organization improvement |
| Tools and technology (For example, quality and comfort of protective equipment) | No measure | Improvement of the availability and comfort of protective equipment |
| Tasks (For example, workload and conflicts with other tasks) | No measure | IPC workflow improvement |
| Internal environment (For example, department layout, number and location of hand hygiene facilities, etc.) | Physical environment improvement | No measure |
| External environment (For example, social respect and understanding, policy support) | No measure | External environment improvement |
| Please choose your most preferred scenario: | I prefer Scenario A | I prefer Scenario B  □ |

**Part two: Personal information**

1 Gender：male female；

2 Age： year；

3 Degree：Doctor Master  Undergraduate  junior college below junior college；

4 Work year： year；

5 Title： No title  Junior  Intermediate  Deputy senior  Deputy；

6 Department：

Date： Name：

**Block two**

**Part one: Preference of intervention**

You can choose the preferred intervention scenario from two alternatives in one set. Each scenario is formulated by the combination of the six attributes in different level. You should compare the six attributes comprehensively and then make your choice. Thirteen sets were formed in this questionnaire.

- **Sample：**

If you choose scenario A, it means that you are more willing to choose scenario A as an intervention to improve your infection prevention and control (IPC) behavior than scenario B.

|  | Scenario A | Scenario B |
| --- | --- | --- |
| Person (For example, IPC knowledge and awareness training) | No measure | Better IPC knowledge and awareness training |
| Organization (For example, attention to IPC of leaders and cultivating a safe atmosphere) | IPC organization improvement | No measure |
| Tools and technology (For example, quality and comfort of protective equipment) | Improvement of the availability and comfort of protective equipment | No measure |
| Tasks (For example, workload and conflicts with other tasks) | No measure | Reduced workload |
| Internal environment (For example, department layout, number and location of hand hygiene facilities, etc.) | Physical environment improvement | No measure |
| External environment (For example, social respect and understanding, policy support) | No measure | External environment improvement |
| Please choose your most preferred scenario: | I prefer Scenario A | I prefer Scenario B  □ |

- **Formal Set**
- Set 1

|  | Scenario A | Scenario B |
| --- | --- | --- |
| Person (For example, IPC knowledge and awareness training) | No measure | Better IPC knowledge and awareness training |
| Organization (For example, attention to IPC of leaders and cultivating a safe atmosphere) | IPC organization improvement | No measure |
| Tools and technology (For example, quality and comfort of protective equipment) | No measure | Improvement of the availability and comfort of protective equipment |
| Tasks (For example, workload and conflicts with other tasks) | Reduced workload | No measure |
| Internal environment (For example, department layout, number and location of hand hygiene facilities, etc.) | No measure | Physical environment improvement |
| External environment (For example, social respect and understanding, policy support) | No measure | External environment improvement |
| Please choose your most preferred scenario: | I prefer Scenario A | I prefer Scenario B  □ |

- Set 2

|  | Scenario A | Scenario B |
| --- | --- | --- |
| Person (For example, IPC knowledge and awareness training) | No measure | Better IPC knowledge and awareness training |
| Organization (For example, attention to IPC of leaders and cultivating a safe atmosphere) | No measure | IPC organization improvement |
| Tools and technology (For example, quality and comfort of protective equipment) | Improvement of the availability and comfort of protective equipment | No measure |
| Tasks (For example, workload and conflicts with other tasks) | Reduced workload | No measure |
| Internal environment (For example, department layout, number and location of hand hygiene facilities, etc.) | No measure | Physical environment improvement |
| External environment (For example, social respect and understanding, policy support) | No measure | External environment improvement |
| Please choose your most preferred scenario: | I prefer Scenario A | I prefer Scenario B  □ |

- Set 3

|  | Scenario A | Scenario B |
| --- | --- | --- |
| Person (For example, IPC knowledge and awareness training) | Better IPC knowledge and awareness training | No measure |
| Organization (For example, attention to IPC of leaders and cultivating a safe atmosphere) | No measure | IPC organization improvement |
| Tools and technology (For example, quality and comfort of protective equipment) | No measure | Improvement of the availability and comfort of protective equipment |
| Tasks (For example, workload and conflicts with other tasks) | IPC workflow improvement | Reduced workload |
| Internal environment (For example, department layout, number and location of hand hygiene facilities, etc.) | Physical environment improvement | No measure |
| External environment (For example, social respect and understanding, policy support) | No measure | External environment improvement |
| Please choose your most preferred scenario: | I prefer Scenario A | I prefer Scenario B  □ |

- Set 4

|  | Scenario A | Scenario B |
| --- | --- | --- |
| Person (For example, IPC knowledge and awareness training) | Better IPC knowledge and awareness training | No measure |
| Organization (For example, attention to IPC of leaders and cultivating a safe atmosphere) | IPC organization improvement | No measure |
| Tools and technology (For example, quality and comfort of protective equipment) | No measure | Improvement of the availability and comfort of protective equipment |
| Tasks (For example, workload and conflicts with other tasks) | No measure | IPC workflow improvement |
| Internal environment (For example, department layout, number and location of hand hygiene facilities, etc.) | Physical environment improvement | No measure |
| External environment (For example, social respect and understanding, policy support) | No measure | External environment improvement |
| Please choose your most preferred scenario: | I prefer Scenario A | I prefer Scenario B  □ |

- Set 5

|  | Scenario A | Scenario B |
| --- | --- | --- |
| Person (For example, IPC knowledge and awareness training) | Better IPC knowledge and awareness training | No measure |
| Organization (For example, attention to IPC of leaders and cultivating a safe atmosphere) | No measure | IPC organization improvement |
| Tools and technology (For example, quality and comfort of protective equipment) | Improvement of the availability and comfort of protective equipment | No measure |
| Tasks (For example, workload and conflicts with other tasks) | Reduced workload | No measure |
| Internal environment (For example, department layout, number and location of hand hygiene facilities, etc.) | Physical environment improvement | No measure |
| External environment (For example, social respect and understanding, policy support) | External environment improvement | No measure |
| Please choose your most preferred scenario: | I prefer Scenario A | I prefer Scenario B  □ |

- Set 6

|  | Scenario A | Scenario B |
| --- | --- | --- |
| Person (For example, IPC knowledge and awareness training) | No measure | Better IPC knowledge and awareness training |
| Organization (For example, attention to IPC of leaders and cultivating a safe atmosphere) | IPC organization improvement | No measure |
| Tools and technology (For example, quality and comfort of protective equipment) | Improvement of the availability and comfort of protective equipment | No measure |
| Tasks (For example, workload and conflicts with other tasks) | No measure | IPC workflow improvement |
| Internal environment (For example, department layout, number and location of hand hygiene facilities, etc.) | Physical environment improvement | No measure |
| External environment (For example, social respect and understanding, policy support) | No measure | External environment improvement |
| Please choose your most preferred scenario: | I prefer Scenario A | I prefer Scenario B  □ |

- Set 7

|  | Scenario A | Scenario B |
| --- | --- | --- |
| Person (For example, IPC knowledge and awareness training) | Better IPC knowledge and awareness training | No measure |
| Organization (For example, attention to IPC of leaders and cultivating a safe atmosphere) | No measure | IPC organization improvement |
| Tools and technology (For example, quality and comfort of protective equipment) | No measure | Improvement of the availability and comfort of protective equipment |
| Tasks (For example, workload and conflicts with other tasks) | Reduced workload | No measure |
| Internal environment (For example, department layout, number and location of hand hygiene facilities, etc.) | No measure | Physical environment improvement |
| External environment (For example, social respect and understanding, policy support) | External environment improvement | No measure |
| Please choose your most preferred scenario: | I prefer Scenario A | I prefer Scenario B  □ |

- Set 8

|  | Scenario A | Scenario B |
| --- | --- | --- |
| Person (For example, IPC knowledge and awareness training) | No measure | Better IPC knowledge and awareness training |
| Organization (For example, attention to IPC of leaders and cultivating a safe atmosphere) | IPC organization improvement | No measure |
| Tools and technology (For example, quality and comfort of protective equipment) | No measure | Improvement of the availability and comfort of protective equipment |
| Tasks (For example, workload and conflicts with other tasks) | IPC workflow improvement | Reduced workload |
| Internal environment (For example, department layout, number and location of hand hygiene facilities, etc.) | No measure | Physical environment improvement |
| External environment (For example, social respect and understanding, policy support) | External environment improvement | No measure |
| Please choose your most preferred scenario: | I prefer Scenario A | I prefer Scenario B  □ |

- Set 9

|  | Scenario A | Scenario B |
| --- | --- | --- |
| Person (For example, IPC knowledge and awareness training) | No measure | Better IPC knowledge and awareness training |
| Organization (For example, attention to IPC of leaders and cultivating a safe atmosphere) | No measure | IPC organization improvement |
| Tools and technology (For example, quality and comfort of protective equipment) | Improvement of the availability and comfort of protective equipment | No measure |
| Tasks (For example, workload and conflicts with other tasks) | No measure | IPC workflow improvement |
| Internal environment (For example, department layout, number and location of hand hygiene facilities, etc.) | No measure | Physical environment improvement |
| External environment (For example, social respect and understanding, policy support) | External environment improvement | No measure |
| Please choose your most preferred scenario: | I prefer Scenario A | I prefer Scenario B  □ |

- Set 10

|  | Scenario A | Scenario B |
| --- | --- | --- |
| Person (For example, IPC knowledge and awareness training) | Better IPC knowledge and awareness training | No measure |
| Organization (For example, attention to IPC of leaders and cultivating a safe atmosphere) | IPC organization improvement | No measure |
| Tools and technology (For example, quality and comfort of protective equipment) | Improvement of the availability and comfort of protective equipment | No measure |
| Tasks (For example, workload and conflicts with other tasks) | No measure | IPC workflow improvement |
| Internal environment (For example, department layout, number and location of hand hygiene facilities, etc.) | No measure | Physical environment improvement |
| External environment (For example, social respect and understanding, policy support) | External environment improvement | No measure |
| Please choose your most preferred scenario: | I prefer Scenario A | I prefer Scenario B  □ |

- Set 11

|  | Scenario A | Scenario B |
| --- | --- | --- |
| Person (For example, IPC knowledge and awareness training) | No measure | Better IPC knowledge and awareness training |
| Organization (For example, attention to IPC of leaders and cultivating a safe atmosphere) | IPC organization improvement | No measure |
| Tools and technology (For example, quality and comfort of protective equipment) | No measure | Improvement of the availability and comfort of protective equipment |
| Tasks (For example, workload and conflicts with other tasks) | IPC workflow improvement | Reduced workload |
| Internal environment (For example, department layout, number and location of hand hygiene facilities, etc.) | Physical environment improvement | No measure |
| External environment (For example, social respect and understanding, policy support) | External environment improvement | No measure |
| Please choose your most preferred scenario: | I prefer Scenario A | I prefer Scenario B  □ |

- Set 12

|  | Scenario A | Scenario B |
| --- | --- | --- |
| Person (For example, IPC knowledge and awareness training) | Better IPC knowledge and awareness training | No measure |
| Organization (For example, attention to IPC of leaders and cultivating a safe atmosphere) | No measure | IPC organization improvement |
| Tools and technology (For example, quality and comfort of protective equipment) | Improvement of the availability and comfort of protective equipment | No measure |
| Tasks (For example, workload and conflicts with other tasks) | IPC workflow improvement | Reduced workload |
| Internal environment (For example, department layout, number and location of hand hygiene facilities, etc.) | Physical environment improvement | No measure |
| External environment (For example, social respect and understanding, policy support) | No measure | External environment improvement |
| Please choose your most preferred scenario: | I prefer Scenario A | I prefer Scenario B  □ |

- Set 13

|  | Scenario A | Scenario B |
| --- | --- | --- |
| Person (For example, IPC knowledge and awareness training) | Better IPC knowledge and awareness training | No measure |
| Organization (For example, attention to IPC of leaders and cultivating a safe atmosphere) | No measure | IPC organization improvement |
| Tools and technology (For example, quality and comfort of protective equipment) | Improvement of the availability and comfort of protective equipment | No measure |
| Tasks (For example, workload and conflicts with other tasks) | Reduced workload | No measure |
| Internal environment (For example, department layout, number and location of hand hygiene facilities, etc.) | Physical environment improvement | No measure |
| External environment (For example, social respect and understanding, policy support) | External environment improvement | No measure |
| Please choose your most preferred scenario: | I prefer Scenario A | I prefer Scenario B  □ |

**Part two: Personal information**

1 Gender：male female；

2 Age： year；

3 Degree：Doctor Master  Undergraduate  junior college below junior college；

4 Work year： year；

5 Title： No title  Junior  Intermediate  Deputy senior  Deputy；

6 Department：

Date： Name：
